# Exploration of Early Biomarkers for Gestational Diabetes Mellitus in Pregnant Women Living Without Obesity

**DOI:** 10.1155/jdr/9762300

**Published:** 2026-07-31

**Authors:** Dan Wu, Meiyi Xu, Cunling Zhang, Shanshan Li, Liying Yao, Mengyuan Geng, Yuling Guo, Zhuo Wei, Wen Li

**Affiliations:** ^1^ Tianjin Institute of Gynecology Obstetrics, Tianjin Central Hospital of Gynecology Obstetrics, Tianjin, China, tjzxfc.com; ^2^ Tianjin Key Laboratory of Human Development and Reproductive Regulation, Tianjin Central Hospital of Gynecology Obstetrics, Tianjin, China, tjzxfc.com; ^3^ Department of Obstetrics, Tianjin Central Hospital of Gynecology Obstetrics, Tianjin, China, tjzxfc.com; ^4^ Department of Clinical Laboratory, Tianjin Central Hospital of Gynecology Obstetrics, Tianjin, China, tjzxfc.com

## Abstract

**Background:**

Early prediction of gestational diabetes mellitus (GDM) enables timely interventions but remains challenging, particularly in women living without obesity who are often overlooked by standard screening methods. This exploratory analysis identifies early pregnancy biomarkers that show associations with GDM up to 8–12 weeks earlier than the established diagnostic timeframe.

**Methods:**

A retrospective nested case‐control study was conducted at Tianjin Central Hospital of Gynecology Obstetrics. After applying the predefined inclusion and exclusion criteria and removing cases with missing blood samples, a total of 136 women were included in the final analysis, comprising 56 women with GDM and 80 non‐GDM controls. These data were used to develop multiple machine learning models, including random forest, logistic regression, SVM, XGBoost, and KNN. The performance of these models was assessed by AUC, sensitivity, specificity, positive predictive value, and negative predictive value in an independent validation set.

**Results:**

The random forest classifier achieved the best performance with an AUC of 0.992 in the validation set, indicating excellent sensitivity and specificity. The model incorporated six measurable variables: age, BMI, and four cytokines (IL‐1*β*, IL‐10, IL‐17A, and IL‐4) participating in immune regulation in pregnancy.

**Conclusions:**

GDM can be predicted with high accuracy early in pregnancy, even among women living without obesity. This study highlights the value of integrating immunological biomarkers with clinical characteristics and machine learning for proactive risk stratification. Although promising, our findings warrant validation in larger, multiethnic cohorts to ensure generalizability.

## 1. Introduction

Gestational diabetes mellitus (GDM) is defined as any degree of glucose intolerance with onset or first recognition during pregnancy [1]. Based on the criteria established by the International Association of Diabetes and Pregnancy Study Groups (IADPSG), the global prevalence of GDM is estimated at 14.0%, making it the most common complication during pregnancy [2]. In mainland China, the overall prevalence of GDM post‐2010 is approximately 15.6% according to IADPSG criteria [3], whereas a 2020 study reported that the prevalence of GDM in northern Chinese cities reached 21% [4].

Pregnant women with GDM face an elevated risk of complications such as preeclampsia, cesarean delivery, labor induction, and premature rupture of membranes [5]. In the long term, these women are more likely to develop Type 2 diabetes [6] and cardiovascular diseases [7]. Likewise, offspring of GDM women face elevated risk of preterm birth, macrosomia, and neonatal complications such as respiratory distress syndrome and hypoglycemia [5]. Furthermore, these children are more likely to develop impaired glucose tolerance [8] and obesity [9] later in life.

Currently, many countries, including China, adopt the WHO 2013 criteria for diagnosing GDM, which recommend a 75‐g, 2‐h oral glucose tolerance test (OGTT) between 24 and 28 weeks of gestation [10]. However, emerging evidence suggests that initiating interventions before 20 weeks of gestation may reduce the composite incidence of adverse neonatal outcomes, such as preterm birth, stillbirth, and respiratory distress [11]. Unfortunately, there is currently no consensus in clinical practice regarding reliable methods for early diagnosis [12]. Several guidelines have recommendations for early screening in the first trimester or at the initiation of antenatal care, which focus on excluding pre‐existing DM in women at high risk [13]. Therefore, an early risk prediction model based on the pathogenesis of GDM may facilitate timely identification and intervention, contributing to improved pregnancy outcomes.

The pathogenesis of GDM is highly complex and multifaceted, including adipose tissue dysfunction, placental hormones or proteins, chronic inflammation, hereditary factors, and so on. Enlarged adipocyte size, followed by tissue inflammation, is widely believed to enhance glucose intolerance by abnormal levels of adipokines such as higher fatty acid–binding protein 4 (FABP4) and leptin (Lep) but lower high molecular weight adiponectin (HMWA) [14]. Although GDM is thought to be associated with overweight and obesity, normal and underweight women are affected as well, particularly in low‐ and middle‐income countries, suggesting that more critical mechanisms remain undiscovered [15]. Other researchers believe that the placenta, which produces a variety of functional proteins and hormones such as Lep, placental growth factor (PlGF), sex hormone‐binding globulin (SHBG), and testosterone (HT), is associated with GDM pathogenesis [16–18]. In addition, it has been demonstrated that placenta‐derived insulin‐like growth factor (IGF), insulin‐like growth factor binding protein 2 (IGFBP2), and PlGF are all associated with GDM [17, 19, 20].

Notably, immune factors such as interleukin‐6 (IL‐6), tumor necrosis factor‐alpha (TNF‐*α*), and immune cells such as regulatory T (Treg) cells are drawing increasing attention and have been proven to be involved in adipose tissue and placental dysfunction, which play a role in maintaining glucose homeostasis [21]. Studies suggest that an increased leukocyte count early in pregnancy is independently and linearly associated with the results of GDM screening tests and the risk of GDM, which may be driven by microbiota [22, 23]. Besides, interleukin‐10 (IL‐10), interleukin‐17 (IL‐17), interferon‐gamma (IFN‐*γ*), and interleukin‐4 (IL‐4) related to T cell function may serve as potential predictive biomarkers for GDM [24–26]. These studies emphasize the importance of inflammation in the pathogenesis of GDM.

Given that the factors described above are implicated in the pathogenesis of GDM or exhibit abnormal levels early in pregnancy, they are theoretically well‐suited to serve as potential predictive biomarkers for GDM. Moreover, most existing models focus on high‐risk populations, often emphasizing obesity as a major risk factor. However, pregnant women living without obesity also develop GDM and are frequently overlooked in early screening. In this study, we analyzed multibiomarkers and clinical data collected before 16 weeks of gestation in women living without obesity. Multiple machine learning models were developed and compared for GDM screening. This approach not only facilitates early identification and preventive intervention but also underscores the value of integrating immune biomarkers to enhance prediction accuracy in this underrepresented group.

## 2. Materials and Methods

### 2.1. Study Population and Clinical Data

This retrospective nested case‐control study was conducted at Tianjin Central Hospital of Gynecology Obstetrics (TJCHGO). A total of 1639 pregnant women who had discharge diagnoses and corresponding blood records from January to December 2023 were initially screened.

To ensure the reliability of baseline data in this small‐sample study, in control group, exclusion criteria for this study included multiple pregnancies; complications such as chronic hypertension, preeclampsia, hyperthyroidism, or other disorders related to metabolic or immune system dysfunction. The GDM group was confirmed according to OGTT results.

For both the GDM and control groups, samples collected after 16 weeks of gestation and samples from participants classified as obese were excluded from the analysis.

All participants provided informed consent, and the study protocol was approved by the Ethics Committee of TJCHGO (Approval No. [2025KY068]). All procedures were conducted in accordance with the Declaration of Helsinki, and patient privacy was strictly protected throughout the study.

### 2.2. Blood Sample Collection and Processing

Peripheral blood samples were randomly obtained from participants under nonfasting conditions at the time of noninvasive prenatal testing conducted at our hospital. Approximately 10 mL of venous blood was aseptically collected from the antecubital vein of each participant into tubes. The samples were centrifuged at 1300 × g for 10 min at 4°C for plasma separation. The plasma was transferred into 1.5‐mL polypropylene microcentrifuge tubes (DNA‐/RNA‐free, Axygen, United States) and stored at −80°C until further analysis. Clinical data, including height, weight, age, and gestational age, were recorded for each participant.

### 2.3. OGTT

According to routine prenatal care guidelines, pregnant women underwent a 75‐g OGTT between 24 and 28 weeks of gestation. In accordance with the criteria established by the IADPSG, GDM was diagnosed if any one of the following plasma glucose values was met or exceeded: fasting glucose ≥ 5.1 mmol/L, 1 − h glucose ≥ 10.0 mmol/L, or 2 − h glucose ≥ 8.5 mmol/L.

### 2.4. Enzyme‐Linked Immunosorbent Assay (ELISA) Analysis

Based on a comprehensive review of the literature, we identified 16 inflammatory, placenta‐derived hormones and proteins, as well as metabolic factors as candidate biomarkers. FABP4 (TW3833), HMWA (TW15935), LEP (TW2945), IGF1R (TW9186), IGFBP2 (TW10656), SHBG (TW4362), HT (TW88745), and PlGF (TW3518) ELISA kits were bought from Shanghai Tongwei Industrial Co., Ltd. TNF‐*α* (88‐7346‐77), IL‐6 (88‐7066‐77), IL‐1*β* (88‐7261‐77), IL‐10 (88‐7106‐77), IL‐17A (88‐7176‐77), IFN‐*γ* (88‐7316‐77), and IL‐4 (88‐7046‐77) ELISA kits were bought from Invitrogen. These biomarkers were measured according to the manufacturers′ instructions.

### 2.5. Statistical Analysis of Baseline Data

Baseline data analysis was conducted using R software (Version 3.6.3). Continuous variables were summarized according to their distribution characteristics. Variables following an approximately normal distribution, as determined by the Shapiro–Wilk test, were expressed as mean ± standard deviation (mean ± SD) and compared using independent samples *t*‐tests. Nonnormally distributed variables were reported as median with interquartile range (median [P25, P75]) and analyzed using the Wilcoxon rank‐sum test (Mann–Whitney *U* test). Categorical variables were summarized as counts and percentages, and group comparisons were conducted using the chi‐square test or Fisher′s exact test when the expected cell count was less than 5. All statistical tests were two‐sided, and a *p* value < 0.05 was considered statistically significant. Data processing and analysis were performed using the *dplyr* (Version 1.1.4) and *stats* (Version 3.6.3) packages in R.

### 2.6. Model Construction and Evaluation

The dataset was randomly divided into a training set and an independent validation set using stratified random sampling to maintain the same proportion of outcome labels in both subsets. Specifically, the createDataPartition function in R was used to generate index numbers for the training set, with 70% of the data allocated for model training and the remaining 30% used for model validation.

Clinical variables, including gestational weeks, age, body mass index (BMI), and inflammatory cytokines measured by ELISA (FABP4, HMWA, LEP, IGF, IGFBP2, SHBG, HT, PlGF, TNF‐*α*, IL‐6, IL‐1*β*, IL‐10, IL‐17A, IFN‐*γ*, and IL‐4), were included as candidate predictors.

To avoid information leakage, variable selection was performed exclusively on the training set using Least Absolute Shrinkage and Selection Operator (LASSO) logistic regression. A fixed random seed was set to ensure reproducibility. The optimal regularization parameter (*λ*) was determined by 10‐fold cross‐validation.

Based on the selected features, five supervised machine learning models were developed: random forest (RF), logistic regression, support vector machine (SVM), Extreme Gradient Boosting (XGBoost), and K‐nearest neighbors (KNN). All models were trained exclusively on the training set. The RF model was implemented using the randomForest package (Version 4.6.14). The logistic regression was performed using the base stats package (Version 3.6.3). The SVM model was constructed with the e1071 package (Version 1.7.16). The XGBoost model was built using the xgboost package (Version 1.7.10.1). The KNN classification was carried out using the class package (Version 7.3.22). The diagnostic performance metrics were calculated using the pROC package (Version 1.18.5).

Model performance was independently evaluated on the validation set. Evaluation metrics included the area under the receiver operating characteristic curve (AUC), sensitivity, specificity, positive predictive value (PPV), and negative predictive value (NPV), with exact 95% confidence intervals computed using the PropCIs package (Version 0.3.0).

## 3. Results

### 3.1. Population Demographics and Clinical Characteristics

**Figure 1:**
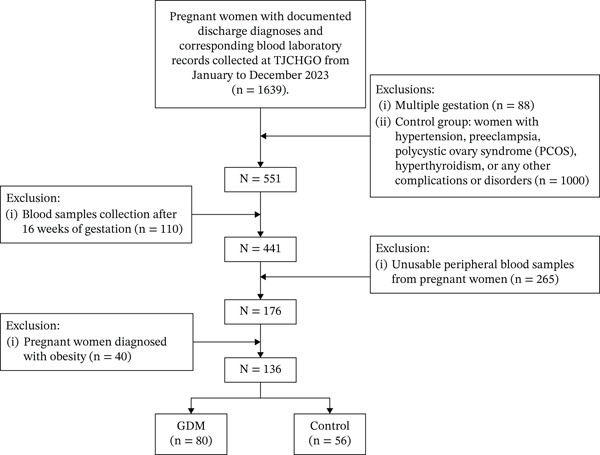
Flowchart of participant inclusion and exclusion.

As shown in Figure 1, based on the predefined inclusion and exclusion criteria, a total of 56 healthy pregnant women and 80 patients with GDM were included in the final analysis. Table 1 summarizes the demographic and clinical characteristics of the participants. The maternal age was 30.11 ± 4.60 years in the control group and 33.17 ± 4.41 years in the GDM group (*p* < 0.001). Gestational age at sampling showed no significant difference between groups (*p* = 0.382). Height was 164.05 ± 4.67 cm and 162.81 ± 4.46 cm in the control and GDM groups, respectively (*p* = 0.123). Weight was 57.49 ± 6.07 kg in the control group and 60.49 ± 7.57 kg in the GDM group (*p* = 0.012). BMI was 21.37 ± 2.14 kg/m^2^ and 22.82 ± 2.70 kg/m^2^ in the control and GDM groups, respectively (*p* < 0.001). Regarding BMI classification, 83.9% (*n* = 47) of the control group and 66.2% (*n* = 53) of the GDM group were classified as living without obesity, whereas 16.1% (*n* = 9) and 33.8% (*n* = 27) were living with obesity, respectively. The distribution differed significantly between groups (*p* = 0.036).

**Table 1:** Clinical parameters of women with and without GDM.

Variables	Control (*n* = 56)	GDM (*n* = 80)	*p* value
Age (years)	30.11 ± 4.60	33.17 ± 4.41	< 0.001
Gestational age (week)	14.64 (14.14, 15.43)	14.64 (14.00, 15.29)	0.382
Height (m)	164.05 ± 4.67	162.81 ± 4.46	0.123
Weight (kg)	57.49 ± 6.07	60.49 ± 7.57	0.012
BMI	21.37 ± 2.14	22.82 ± 2.70	< 0.001
Living without obesity (%)	47 (83.9)	53 (66.2)	0.036
Living with obesity (%)	9 (16.1)	27 (33.8)	—
Gestational age at delivery (weeks)	39.3 ± 0.96	38.3 ± 2.55	0.002
OGTT—fasting plasma glucose (mmol/L)	4.44 ± 0.33	4.81 ± 0.53	< 0.001
OGTT—1‐h plasma glucose (mmol/L)	7.31 ± 1.54	9.72 ± 1.49	< 0.001
OGTT—2‐h plasma glucose (mmol/L)	6.55 ± 1.17	8.41 ± 1.8	< 0.001

*Note:* Continuous variables were described as mean ± SD if normally distributed (assessed by the Shapiro–Wilk test) and compared using independent *t*‐tests; otherwise, they were expressed as median (P25, P75) and compared using the Wilcoxon rank‐sum test (Mann–Whitney *U* test). Categorical variables were presented as counts (percentages) and compared using the chi‐square test or Fisher′s exact test when the expected count was < 5. All tests were two‐sided, and *p* < 0.05 was considered statistically significant.

As shown in Table 2, among the 16 measured biomarkers, 6 showed statistically significant differences between the two groups (*p* < 0.05), including IL‐1*β*, IL‐10, IL‐17, IFN‐*γ*, IL‐4, and PlGF. The remaining 10 proteins did not differ significantly between groups. Comparisons among the two groups were evaluated using Wilcoxon rank‐sum tests, contingent upon the normality of their distribution, which was assessed via the Shapiro–Wilk test. ELISA raw data are provided in Table S1.

**Table 2:** Plasma proinflammatory cytokine concentrations in participants with and without GDM.

Biomarker	Control (*n* = 56)	GDM (*n* = 80)	*p*value
TNF‐*α* (pg/mL)	29.05 (24.75, 35.44)	26.34 (24.23, 30.77)	0.063
IL‐6 (pg/mL)	5.05 (4.58, 5.74)	5.15 (4.80, 5.78)	0.153
IL‐1*β* (pg/mL)	1.53 (1.28, 1.83)	1.84 (1.66, 2.07)	< 0.001
IL‐10 (pg/mL)	4.22 (3.82, 4.73)	5.03 (4.63, 5.48)	< 0.001
IL‐17 (pg/mL)	6.58 (6.24, 7.34)	8.22 (7.48, 9.04)	< 0.001
IFN‐*γ* (pg/mL)	55.55 (52.07, 61.87)	61.91 (56.90, 68.18)	< 0.001
IL‐4 (pg/mL)	3.50 (3.27, 3.82)	4.09 (3.71, 4.47)	< 0.001
FABP4 (ng/mL)	552.86 (461.98, 791.04)	532.19 (454.03, 728.92)	0.611
HMWA (pg/mL)	106.15 (93.69, 147.75)	100.92 (80.14, 132.82)	0.084
HT (nmol/L)	3.61 (3.11, 5.33)	4.30 (3.32, 6.09)	0.182
IGF (*μ*g/L)	23.30 (19.84, 29.71)	21.96 (18.82, 30.32)	0.665
IGFBP2 (ng/mL)	5.62 (4.70, 7.66)	5.40 (4.15, 7.40)	0.360
Lep (*μ*g/L)	4.01 (3.25, 5.13)	4.34 (3.45, 5.91)	0.213
PlGF (ng/L)	48.47 (40.55, 62.00)	62.23 (52.22, 93.60)	< 0.001
SHBG (ng/L)	16.92 (13.97, 22.46)	17.81 (14.69, 24.12)	0.539

*Note:* The results of ELISA were described as mean ± SD if normally distributed (assessed by the Shapiro–Wilk test) and compared using independent t‐tests; otherwise, they were expressed as median (P25, P75) and compared using the Wilcoxon rank‐sum test (Mann–Whitney *U* test).

### 3.2. Feature Selection and Construction of Five Prediction Models

**Figure 2:**
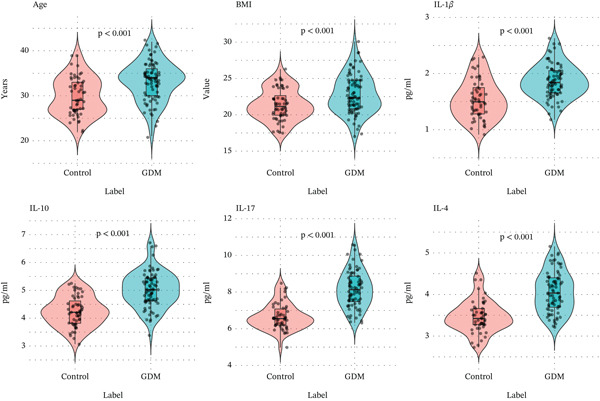
Comparison of six selected features (age, BMI, IL‐1β, IL‐10, IL‐17, and IL‐4) between the control and GDM groups. These features were identified through LASSO regression.

**Figure 3:**
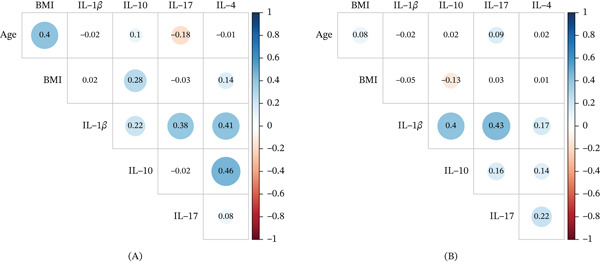
Correlation heatmaps of the six selected features—age, BMI, IL‐1*β*, IL‐10, IL‐17, and IL‐4—in (A), the control group and (B) the GDM group.

We applied the LASSO regression method to select features from ELISA assay results combined with clinical information (age, BMI, and gestational age). After performing 10‐fold cross‐validation, six factors—age, BMI, IL‐1*β*, IL‐10, IL‐17, and IL‐4—were selected for model construction. To avoid data leakage, the feature selection process using LASSO was conducted exclusively within the training set. The differences in these indicators between the control group and the GDM group are shown in Figure 2, all these factors showed significant differences between the control group and the GDM group (*p* < 0.05). Figure 3 presents the correlation heatmaps of the six selected features. Panel (A) shows the control group, and Panel (B) shows the GDM group. In the control group, BMI was moderately correlated with age (*r* = 0.40). Moderate correlations were also observed between IL‐1*β* and IL‐17 (*r* = 0.38), IL‐1*β* and IL‐4 (*r* = 0.41), as well as IL‐10 and IL‐4 (*r* = 0.46). In the GDM group, IL‐1*β* showed moderate correlations with IL‐10 (*r* = 0.40) and IL‐17 (*r* = 0.43).

We evaluated five machine learning models—RF, logistic regression, SVM, XGBoost, and KNN—for predicting the outcome using the selected six features (“age,” “BMI,” “IL‐1*β*,” “IL‐10,” “IL‐17,” and “IL‐4”). Model training was performed on the training set, and performance was assessed on an independent validation set.

**Figure 4:**
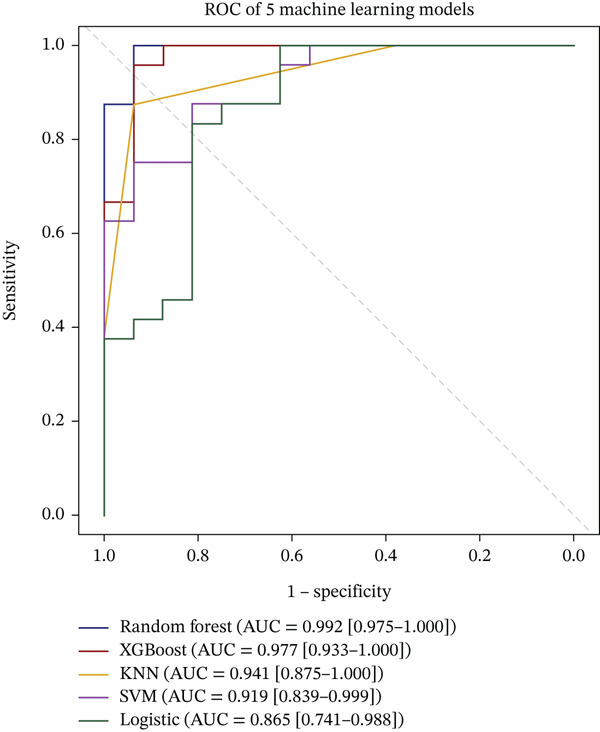
ROC curves of 5 machine learning models.

As shown in Figure 4 and Table 3, the RF model achieved the highest predictive performance with an AUC of 0.992 (95% CI: 0.975–1.000), followed by XGBoost with an AUC of 0.977 (95% CI: 0.933–1.000), KNN with an AUC of 0.941 (95% CI: 0.875–1.000), SVM with an AUC of 0.919 (95% CI: 0.839–0.999), and logistic regression with an AUC of 0.865 (95% CI: 0.741–0.988).

**Table 3:** Performance metrics of five machine learning models.

Model	AUC (95% CI)	Sensitivity (95% CI)	Specificity (95% CI)	PPV (95% CI)	NPV (95% CI)
Random forest	0.992 (0.975–1.000)	1.000 (0.858–1.000)	0.875 (0.617–0.984)	0.923 (0.749–0.991)	1.000 (0.768–1.000)
XGBoost	0.977 (0.933–1.000)	1.000 (0.858–1.000)	0.813 (0.544–0.960)	0.889 (0.708–0.976)	1.000 (0.753–1.000)
KNN	0.941 (0.875–1.000)	0.958 (0.789–0.999)	0.563 (0.299–0.802)	0.767 (0.577–0.901)	0.900 (0.555–0.997)
SVM	0.919 (0.839–0.999)	1.000 (0.858–1.000)	0.563 (0.299–0.802)	0.774 (0.589–0.904)	1.000 (0.664–1.000)
Logistic regression	0.865 (0.741–0.988)	1.000 (0.858–1.000)	0.563 (0.299–0.802)	0.774 (0.589–0.904)	1.000 (0.664–1.000)

In terms of sensitivity, all models except KNN achieved perfect sensitivity of 1.00, whereas KNN reached 0.958 (95% CI: 0.789–0.999). Specificity varied across models, with RF achieving the highest specificity of 0.875 (95% CI: 0.617–0.984), followed by XGBoost at 0.813 (95% CI: 0.544–0.960). Logistic regression, SVM, and KNN had lower specificities of approximately 0.56.

PPVs ranged from 0.77 to 0.92, with RF and XGBoost demonstrating superior PPV (0.92 and 0.89, respectively). NPVs were high across all models, exceeding 0.90. Figure 3 shows the ROC curves of all five models along with their corresponding AUC values and 95% confidence intervals. Table 3 presents the AUC, sensitivity, specificity, PPV, and NPV values of all models along with their 95% confidence intervals.

**Figure 5:**
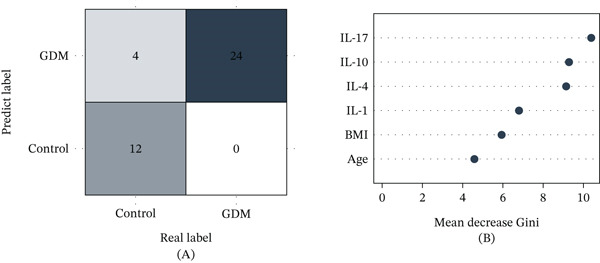
(A) Confusion matrix of the random forest model on the validation set. (B) Mean decreases in Gini index for the six selected features in the random forest model.

Overall, the RF model showed the best balance of sensitivity and specificity, making it the most robust classifier among the five tested models. Figure 5A shows the confusion matrix of the RF model predicting the validation set. Figure 5B displays the importance of six predictive features in the RF model, ranked by mean decrease in Gini index. The importance ranking is as follows: IL‐17, IL‐0, IL‐4, IL‐1*β*, BMI, and age.

## 4. Discussion

GDM is the most prevalent complication of pregnancy, carrying significant short‐ and long‐term consequences for mothers, babies, and even subsequent generations. Recently, experts are urging a paradigm shift toward earlier diagnosis and treatment. Mounting evidence demonstrates that initiating interventions—especially before 14 weeks′ gestation—markedly lowers neonatal morbidity, reduces perineal trauma, and increases breastfeeding initiation, thereby curbing the long‐term risks of obesity and Type 2 diabetes for both mother and child [11]. The conventional strategy of screening at 24–28 weeks misses a substantial proportion of women who would benefit from timely care. Integrating early screening into routine antenatal services is therefore imperative to identify and manage GDM sooner, alleviating its growing global burden.

A multitude of early prediction models have already emerged. Initially, and predominantly, the prediction of GDM primarily relied on the testing results of glucose, insulin, and clinical information yield modest to strong performance according to different criteria. Bayesian model averaging (BMA) approach with age and BMI at booking predicted GDM with an AUC of 0.71 [27]. Adding insulin physiology (PlP) index was able to lift GDM‐prediction AUC from 0.70 (95% CI 0.61–0.79) to 0.87 (0.80–0.93), independent of baseline characteristics [28]. High fasting (AUC: 0.68) and 1‐h OGTT plasma glucose concentrations at 15 weeks′ gestation (AUC: 0.71) were also able to predict GDM [11, 29, 30]. Besides, HbA1c measured at a median 47 days′ gestation was also proven 98.4% (95% CI 97–99.9%) specific for GDM (PPV = 52.9*%*) prediction [31]. Yet, conventional wisdom holds that in the second trimester, fasting glucose remains low despite rising placental hormones; any marked elevation of glucose, insulin, or HbA1c therefore points to previously undiagnosed pre‐existing diabetes.

Despite the limited predictive power of the models in the studies described above, they converge on a key insight: GDM can be anticipated well before traditional screening windows, and performance should improve as richer clinical data or more sensitive biomarkers are incorporated. Indeed, a growing body of evidence reveals that metabolic pathways are already perturbed in early pregnancy, prompting the development of prediction algorithms anchored in metabolic signatures. A lipidomic study performed before 16 weeks′ gestation showed that lipid biomarkers raised the AUC for GDM prediction beyond age and BMI alone from 0.69 to 0.74 [32]. Similarly, readily measurable metabolic proteins—FABP4, adiponectin, IGF, IGFBP2, and Lep—have been implicated in glucose homeostasis and proposed as accessible early‐pregnancy markers [14, 33, 34].

However, results in this study exhibited no significant differences in plasma FABP4 (*p* = 0.611), HMWA (*p* = 0.084), IGF (*p* = 0.665), IGFBP2 (*p* = 0.360), or Lep (*p* = 0.213) measured before 16 weeks between women who later developed GDM and those who did not. This discrepancy is because our participants were predominantly nonobese; these biomarkers are most strongly associated with adipose‐tissue dysfunction, a condition far more prevalent in individuals with elevated BMI [34–36].

Beyond metabolic fingerprints, a growing literature highlights early‐pregnancy perturbations in placenta‐derived factors among women who later develop GDM. In a prospective study of 837 women, adding first‐trimester serum IGFBP‐1 to a clinical model (maternal age, gravidity, family history of diabetes, BMI, and gestational week at sampling) raised the AUC for GDM prediction from 0.66 to 0.72 (*p* = 0.008) [37]. Cell‐free DNA and RNA are rapidly emerging as noninvasive sentinels of placental function: a retrospective cohort of 3200 pregnancies showed that whole‐genome promoter profiling of circulating DNA achieved 72.1% accuracy for GDM detection [38]. Complementing these data, placenta‐derived PlGF, SHBG, and HT have all been reported to differ in women destined to develop GDM [18, 39, 40].

In this study, we found a significant different in PlGF (*p* < 0.001) but not HT (*p* = 0.182) or SHBG (*p* = 0.539) among GDM and non‐GDM group. This discrepancy is best explained by the biology of each marker: PlGF is specific secreted by placenta, direct link to angiogenesis, and steep early‐gestation rise—emerges as a sensitive early GDM marker. By contrast, HT and SHBG are positively associated with maternal BMI and maternal age, whereas we exclude participants with elevated values for both parameters, attenuating their discriminative power for GDM [41].

In recent years, the crosstalk between metabolism and immunity/inflammation has garnered increasing attention. Recently, cohort studies showed high systemic inflammation response index (SIRI), systemic immune‐inflammation index (SII), neutrophil‐to‐lymphocyte ratio (NLR) and aggregate systemic inflammatory response index (AISI) measured at first trimester predicted subsequent GDM at 24–28 weeks [42–44]. Besides, abnormal inflammatory cytokines like IL‐6, Il‐1*β*, TNF‐*α*, IL‐10, IL‐17, IFN‐*γ*, and IL‐4 were widely studied and have been proven predicting subsequent GDM [22, 45, 46].

Here, we have found significantly IL‐1*β*, IL‐10, Il‐17, IFN‐*γ*, IL‐4 differences between the two groups (*p* < 0.001), but not TNF‐*α* (*p* = 0.063) or IL‐6 (*p* = 0.153). These data pinpoint the decisive distinction between the two groups not in the amplitude of acute inflammation, but in the polarization and regulatory balance of the adaptive immune response—specifically, the Th1/Th2/Th17/Treg axis. This aligns with mounting evidence underscoring the pivotal role of this axis in GDM pathogenesis [47–50]. Future investigations should therefore prioritize functional reprogramming within this axis and its direct clinical ramifications.

Recently, machine learning models have demonstrated remarkable power in predicting GDM. A LightGBM model using retrospective data from 588,622 pregnancies in Israel achieved high accuracy in predicting GDM (AUC = 0.85), significantly outperforming a baseline risk score (AUC = 0.68) [51]. A cohort study including 394 participants developed a XGBoost model predicted GDM by clinical data, microbiome, and cytokines also with very high accuracy (AUC = 0.83) [22]. These studies highlight the advantages of different machine learning models in GDM prediction.

In the present study, we developed and validated several such models that incorporate six readily accessible variables—age, BMI, and first‐trimester (< 16 weeks) plasma levels of IL‐1*β*, IL‐10, IL‐17A, and IL‐4—each established as pivotal in GDM pathogenesis. Notably, the RF classifier achieved the highest diagnostic performance, with an AUC of 0.992, demonstrating excellent sensitivity and specificity. Although logistic regression also performed reasonably well, ensemble methods such as RF and XGBoost showed superior discriminative ability (AUC > 0.95). This discrepancy likely reflects the ability of tree‐based models to capture nonlinear interactions and complex relationships among features.

The high prediction accuracy may promote a unique opportunity for proactive intervention, lifestyle modification, and individualized prenatal care to improve both maternal and fetal outcomes in pregnant women living without obesity, an underrepresented group in previous research.

However, this study has several limitations. Due to the presence of selection bias, all results should be interpreted with great caution. The sample size was moderate and drawn from a single center and a single race, which may limit generalizability. The reported predictive values, including PPV and NPV, are specific to this selected sample and model and may not directly apply to the general population.

External validation in larger, multiethnic cohorts is essential to confirm the robustness of our results. Additionally, although our model used early biomarkers, it did not include genetic, metabolic, or lifestyle variables, which could potentially enhance predictive performance.

## 5. Conclusion

In summary, this exploratory study suggests that integrating maternal clinical characteristics with selected inflammatory biomarkers collected before 16 weeks of gestation may aid in the early identification of women at risk for GDM. Although the machine learning models showed encouraging performance in this cohort of women without obesity, the findings should be interpreted with caution due to the small sample size and potential selection bias. Larger, prospective, and multicenter studies are needed to validate these preliminary results and determine their applicability in broader clinical settings.

## Author Contributions

Dan Wu, Meiyi Xu, and Cunling Zhang contributed equally to this work.

## Funding

This study was supported by the Tianjin Health Research Project (TJWJ2023QN067 to Meiyi Xu, TJWJ2023QN072 to Cunling Zhang, and TJWJ2026QN080 to Dan Wu).

## Conflicts of Interest

The authors declare no conflicts of interest.

## Supporting information


**Supporting Information** Additional supporting information can be found online in the Supporting Information section. Table S1: Clinical data and calculated concentrations for each plasma sample obtained from the ELISA assay.

## Data Availability

Data are available in the article′s Supporting Information.
